# Bridging the gap from medical to psychological safety assessment: consensus study in a digital mental health context

**DOI:** 10.1192/bjo.2024.713

**Published:** 2024-06-03

**Authors:** Rayan Taher, Palak Bhanushali, Stephanie Allan, Mario Alvarez-Jimenez, Heather Bolton, Laura Dennison, Brian E. Wallace, Heather D. Hadjistavropoulos, Charlotte L. Hall, Amy Hardy, Alasdair L. Henry, Sam Lane, Tess Maguire, Adam Moreton, Talar R. Moukhtarian, Elvira Perez Vallejos, Sukhi Shergill, Daniel Stahl, Graham R. Thew, Ladislav Timulak, David van den Berg, Noemi Viganò, Ben Wensley Stock, Katherine S. Young, Jenny Yiend

**Affiliations:** Department of Psychosis Studies, Institute of Psychiatry, Psychology & Neuroscience, King's College London, UK; Institute of Health and Wellbeing, University of Glasgow, UK; Centre for Youth Mental Health, University of Melbourne, Australia; Orygen, Parkville, Australia; Unmind, London, UK; School of Psychology, University of Southampton, UK; Calmsie, Warsaw, Poland; Department of Psychology, University of Regina, Canada; NIHR MindTech-MedTech Co-operative, NIHR Nottingham Biomedical Research Centre, School of Medicine, Institute of Mental Health, University of Nottingham, UK; Big Health Ltd, London, UK; SilverCloud by Amwell, Boston, USA; ORCHA, Daresbury, UK; Mental Health and Wellbeing Unit, Warwick Medical School, University of Warwick, UK; Kent and Medway Medical School, Canterbury, UK; Department of Biostatistics and Health Informatics, Institute of Psychiatry, Psychology & Neuroscience, King's College London, UK; Department of Experimental Psychology, University of Oxford, UK; Oxford Health NHS Foundation Trust, Oxford, UK; School of Psychology, Trinity College Dublin, Ireland; Department of Clinical Psychology, VU University and Amsterdam Public Health Research, Amsterdam, Netherlands; University of Oxford Medical Sciences Division, University of Oxford, UK; Social Genetic and Developmental Psychiatry Centre, Institute of Psychiatry, Psychology & Neuroscience, King's College London, UK

**Keywords:** Digital mental health interventions, mental health, safety, MHRA, consensus

## Abstract

**Background:**

Digital Mental Health Interventions (DMHIs) that meet the definition of a medical device are regulated by the Medicines and Healthcare products Regulatory Agency (MHRA) in the UK. The MHRA uses procedures that were originally developed for pharmaceuticals to assess the safety of DMHIs. There is recognition that this may not be ideal, as is evident by an ongoing consultation for reform led by the MHRA and the National Institute for Health and Care Excellence.

**Aims:**

The aim of this study was to generate an experts’ consensus on how the medical regulatory method used for assessing safety could best be adapted for DMHIs.

**Method:**

An online Delphi study containing three rounds was conducted with an international panel of 20 experts with experience/knowledge in the field of UK digital mental health.

**Results:**

Sixty-four items were generated, of which 41 achieved consensus (64%). Consensus emerged around ten recommendations, falling into five main themes: Enhancing the quality of adverse events data in DMHIs; Re-defining serious adverse events for DMHIs; Reassessing short-term symptom deterioration in psychological interventions as a therapeutic risk; Maximising the benefit of the Yellow Card Scheme; and Developing a harmonised approach for assessing the safety of psychological interventions in general.

**Conclusion:**

The implementation of the recommendations provided by this consensus could improve the assessment of safety of DMHIs, making them more effective in detecting and mitigating risk.

Digital Mental Health Interventions (DMHIs) are interventions that use technologies such as apps, computers or virtual reality to support people living with mental health conditions.^1,2^ Such interventions have the potential to reach a large number of individuals, significantly widen access to care and effectively scale evidence-based support.^1^ There is increasing evidence for the effectiveness of DMHIs in improving mental health conditions; however, evaluating their safety remains a key challenge for professionals in the field.^1,3,4^

DMHIs that meet the definition of a ‘medical device’ are regulated by various medical regulatory bodies across the world.^5,6^ A medical device, as defined by the EU Directive 93/42/EEC.11, is ‘any instrument, apparatus, appliance, software, material or other article, whether used alone or in combination, including the software intended by its manufacturer to be used specifically for diagnostic and/or therapeutic purposes and necessary for its proper application, intended by the manufacturer to be used for human beings for the purpose of diagnosis, prevention, monitoring, treatment or alleviation of disease’.^7^ In the UK, the Medicines and Healthcare products Regulatory Agency (MHRA) is responsible for regulating both pharmaceuticals and medical devices, and thus all DMHIs are classified as such.^6^ Therefore the UK's regulatory framework currently applies only to DMHIs intended to treat or alleviate mental ill health, for example those designed for specific, clinically recognised conditions like depression, paranoia or insomnia and specifically intended for use only in those predefined populations. In contrast there is a wide range of similar products that are intended to improve mental well-being but which fall outside the medical regulatory framework and therefore the remit of this paper.^6,8^ This includes anything designed for general public use, for example wellness, exercise, healthy eating, meditation/mindfulness or motivational apps.

From a safety perspective, a medical device's life cycle can be divided into two phases: the pre- and post-market phase. The pre-market phase is when the product's effectiveness and safety is still being tested. The post-market phase is when the product has been approved by the appropriate regulators to be placed on the market and used publicly.^6^

## Assessing the safety of DMHIs in the pre-market phase

In the pre-market phase, the safety of DMHIs is assessed mainly in clinical trials by collecting and analysing Adverse Events and Serious Adverse Events (SAEs) data.^9^ According to the Good Clinical Practice guidelines of the International Council for Harmonization, an adverse event is an ‘untoward medical occurrence’, and an SAE is an adverse event that results in hospitalisation, death, disability or a birth defect, is a medically important event or would have resulted in any of these outcomes if preventative action had not been taken.^1^

Negative effects or adverse events relevant to mental health interventions include deterioration of symptoms (the ‘deterioration effect’), novel symptoms and/or non-response.^10^ The deterioration effect occurs when people's symptoms worsen during therapy.^11^ Approximately 3–10% of psychotherapy patients experience deterioration.^11^ There is division in the literature on whether symptom deterioration is an integral and necessary part of therapy or whether it is an unwanted side-effect.^12^ Novel symptoms are when people experience new mental health symptoms during treatment, ones that they did not present with prior to the onset of the treatment.^13^ Non-response is when people's target symptoms do not improve. This is seen as a negative effect because the treatment might have prevented the person from receiving an effective therapy.^13^ Furthermore, the digital aspect of DMHIs introduces new risks to mental health therapies such as technical issues and privacy concerns.^1,14^

Despite efforts to adequately assess the safety of mental health interventions, numerous research studies have found that the collection, monitoring and analysis of adverse events and SAEs data in face-to-face/digital psychological therapy trials are still in a premature phase.^4,12–15^ A recent review on the identification and categorisation of adverse events in DMHI trials found that only six out of 23 of the included trials (26%) reported adverse events data within their primary results.^1^ The lack of consensus on the definition of what constitutes an adverse event and SAE in a trial for a mental health intervention and what data needs to be collected, and how, have been cited as the main reasons for the current inferior standard of adverse events monitoring and reporting in DMHIs.^1,4,16^ Furthermore, reliance on guidelines that were originally developed for assessing the safety of pharmacotherapies can be a further hindrance.^1^

## Assessing the safety of DMHIs in the post-market phase

In the post-market phase, the MHRA uses Post Market Surveillance (PMS) to monitor the safety of drugs and medical devices that reach the market.^17^ As part of PMS, the manufacturer is responsible for continuously monitoring the safety of their product(s), implementing strategies to minimise risk(s) and reporting their findings to the regulatory body.^7^ Additionally, the MHRA uses a spontaneous recording system called the ‘Yellow Card Scheme’ which is a safety monitoring system.^17^ Its purpose is to identify new side-effects that might not have been previously known and to monitor the safety of products on the market. It allows health professionals, as well as patients and caregivers, to report any suspected adverse reactions directly to the MHRA.^17^ The Yellow Card Scheme has shown effectiveness in helping the MHRA assess the safety of drugs and medical devices.^17^ A review of Yellow Card data found that, compared with health professionals, patients are more likely to report adverse reactions that fall under ‘psychiatric disorders’ – a categorisation that groups all psychiatric, psychological and mental health reactions under the Yellow Card Scheme.^18^ It is speculated that this might be because health professionals may give less importance to such reactions (mental health reactions), or they do not acknowledge such reactions as ‘adverse’.^18^ This further highlights the importance of adapting and promoting the use of the Yellow Card Scheme to assess the safety of DMHIs.

The regulation of DMHIs using medical regulatory methods has some benefits, such as integrating mental health interventions into an established and robust regulatory framework used across the healthcare sector.^5^ However, using a regulatory process for DMHIs that was originally developed for other purposes could have several undesirable consequences, such as missing important harms or overemphasising less significant harms.^4^ There is already an awareness of the need for better regulatory methods for DMHIs; in the UK, this is evident from the £1.8 million received by the MHRA and National Institute for Health and Care Excellence to specifically explore the regulation of DMHIs.^19^ To contribute towards this objective, we employed the Delphi technique to reach an experts’ consensus on ‘What are the adaptations needed for the existing medical based model that is used to assess safety of DMHIs?’

## Method

### Study design

An online Delphi technique was used to reach an experts’ consensus on how the medical methods used for assessing safety can be adapted to better meet the objectives of DMHIs. The Delphi technique is a tool used to reach a consensus, through controlled feedback on topics with limited evidence or where a consensus does not exist.^20,21^ The main components of a Delphi technique are anonymity, iteration, controlled feedback and statistical stability.^13,21,22^

### Recruitment and participants

Purposive sampling was used to invite participants to participate in this Delphi study. Individuals were eligible to participate if they were professionals (researchers, clinicians or regulatory consultants) with experience/knowledge in the field of digital mental health in the UK. There were no exclusion criteria. Experts who met the eligibility criteria were contacted via email or LinkedIn. A snowball technique was also used to recruit participants. Thirty-six professionals were invited to join the panel, of whom 25 consented (69%) and 20 participated (55%). Thus, the sample size was in the average range for Delphi studies.^20^ Of the 20 participants, 11 (55%) were females. The majority of the participants (17/20, 85%) were based in the UK, with three living in Canada, Australia and the Netherlands. Participants had an average of 19 years’ (range 5–37, s.d. = 7.45) general working experience and 8 years (range 2–15, s.d. = 4.51) in the field of digital mental health. See Table 1 for further details on the participants’ occupations. As common in expert Delphi studies, participating panel members were offered authorship on any resulting publications.^23^

**Table 1:** Experts’ occupations and employment sector

	Number	Percentage
Experts’ occupation		
Academic	7	35
Clinical academic	7	35
Clinician	4	20
Regulatory consultant	1	5
Researcher in the industry (doctoral level)	1	5
Experts’ sectors of employment		
Higher education	13	65
Industry (private sector)	5	25
Regulatory board	2	10

### Data collection and analysis

Three rounds were conducted to reach a consensus in this study. Participants were given 2 weeks to complete each round.

#### Round 1: item generation

Participants were sent a link to an 11-question qualitative questionnaire to collect their opinion on how the safety of DMHIs is currently assessed (see Supplementary Appendix A available at https://doi.org/10.1192/bjo.2024.713 for the list of questions). Open-ended qualitative questionnaires are usually used in Delphi studies to generate items using the panel's opinions.^20^ Twenty participants completed round 1. The qualitative data collected were analysed using open coding to generate items. Open coding is ‘the process of identifying, naming, and categorising concepts in the data’.^24^ The authors R.T. and P.B. analysed the data separately and generated items based on the data. Afterwards, they compared and discussed their individual items and agreed on a final list of 64 items. This process was overseen by a third author J.Y.

#### Round 2: collecting controlled feedback

A quantitative questionnaire comprising the 64 final items was shared with all 20 participants who participated in round 1. Participants were asked to rate their agreement with each item on a 5-point Likert scale (strongly disagree, disagree, neutral, agree or strongly agree). Twenty participants completed round 2 (100%).

#### Round 3: reaching a quantitively predefined consensus

**Fig. 1:**
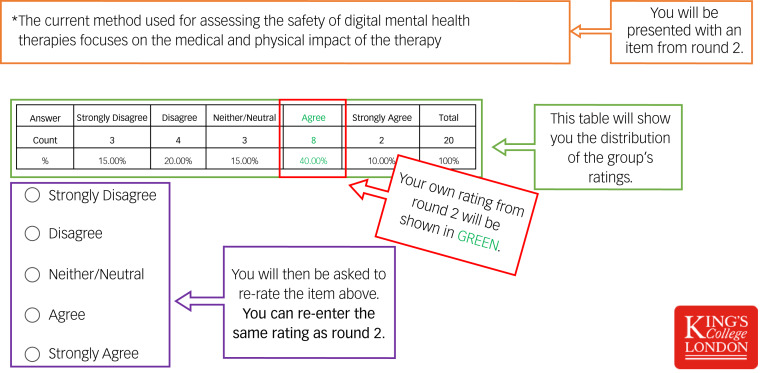
A screenshot of the guide used to assist participants in round 3.

Twenty individual questionnaires were developed. Each questionnaire contained the same items and rating options from round 2, the group's answers, plus the individual's rating. Considering the group's ratings, participants were given an opportunity to reappraise their previous ratings (see Fig. 1). Eighteen participants (90%) completed round 3. The percentage of people who agreed (agree/strongly agree) and disagreed (disagree/strongly disagree) with each item was calculated. Consensus was predefined as any item that reached 80% agreement (the most common method used to define consensus.^25,26^

### Ethical clearance

This study was registered as a minimal risk study at King's College London under the reference number MRSP-22/23-34777. Under the above approval, consent was implied by participants’ email agreement to take part and completion of the various requested survey activities (Round 1–3). Furthermore, at the end of each online questionnaire, the following message was presented to participants: ‘Please be aware that submitting this form implies consent to participate in this research’.

## Results

Forty-one out of 64 items reached consensus (64%). The items that reached consensus in this study can be grouped under five main themes. Table 2 shows percent agreement for the 41 items reaching consensus, organised by theme. Supplementary Appendix B shows all 64 items with corresponding agreement and disagreement rates.

**Table 2:** Items from round 1 reaching consensus (≧80 agreement) organised by theme. Bold text denotes items scoring >90% agreement

Theme	Generated item (percentage of agreement)
Enhancing the quality of adverse events data in Digital Mental Health Interventions (DMHIs)	**Generating publication guidelines for reporting adverse events data that are required by journals can help ensure that adverse events data are collected and reported consistently**. (100%)
	**Determining the relatedness of the adverse event to the therapy is paramount for assessing safety**. (94%)
	**The digital mental health field lacks clear guidance on what adverse events data should be collected, when and how this should happen, and how these should be categorised, analysed and reported**. (94%)
	When assessing adverse events in DMHIs, it is very important to differentiate between self-guided and clinician-guided therapies. (89%)
Redefining serious adverse events for DMHIs	**In the context of mental health therapies, it is important for ‘serious’ to be inclusive of serious effects on mood, behaviour, general well-being and functioning, not just physical harms**. (100%)
	**Increase in harm towards others.** (100%)
	**Increase in intentional self-harm.** (100%)
	**Seizures induced because of on-screen flashes.** (100%)
	**A suicide attempt.** (94%)
	**The current definition of serious adverse events (hospitalisation, death, congenital abnormalities …) is not fit for purpose for mental health interventions compared with pharmaceutical, surgical etc.** (94%)
	Any harm that is sustained for longer than 6 months. (89%)
	Congenital abnormalities as a serious adverse event of digital mental health intervention is not plausible. (83%)
Reassessing short-term deterioration in face-to-face and DMHIs as a therapeutic risk	**Discuss a clear rationale for each therapy activity/exercise.** (100%)
	**People need to be informed about short-term symptom deterioration/distress (through the process of informed consent). In a digital context, this can be replicated through text, audio or video content.** (94%)
	**It is important for therapists to normalise symptom deterioration. In a digital context, this can happen as part of a discussion with a trained mental health professional or via the content of the digital therapy.** (94%)
	**Develop and maintain a strong therapeutic alliance.** (94%)
	**Help the individual think about sources of support that they can use between sessions if needed. In a digital context, provide people with alternative sources of support should they need it.** (94%)
	**Provide individuals with information on how to manage symptom deterioration.** (94%)
	The short-term distress generated by focusing on the emotional experience (perhaps discussing painful or traumatic experiences or going into new or anxiety-provoking situations) is a part of the therapy process and should not be considered an adverse event or safety concern. (89%)
	Long-term follow-ups are needed to differentiate between short-term and long-term deterioration. (89%)
	Short-term deterioration is a momentary side-effect of therapy and one that should be monitored, so that negative outcomes/harm do not result from the treatment. (83%)
	Monitoring of symptoms through regular symptom checks could also mitigate the risks of deterioration. (83%)
	DMHIs should have pre-set thresholds of deterioration that trigger an escalation such as a face-to-face intervention to mitigate risk. (83%)
Maximising the benefit of the Yellow Card Scheme in DMHIs	**Intentional self-harm.** (100%)
	**Threat to others**. (100%)
	**There is not enough awareness among individuals that the Yellow Card System is used for reporting adverse reactions in digital mental health therapies**. (100%)
	**Grouping mental health adverse events under ‘psychiatric disorders’ may discourage reporting milder mental health adverse events. Thus, using a broader category such as ‘mental health adverse events’ is more appropriate.** (94%)
	**Severity/impact on the individual.** (94%)
	**A key problem seems to be that it relies on individuals to report risks, which they may not be willing or able to do. To mitigate this, Patient Reported Outcomes could be automatically collected within the digital therapy itself.** (94%)
	**Suicidal ideation/behaviour.** (94%)
	Grouping all mental health adverse reactions under ‘psychiatric disorders’ risks losing useful and important detail on the different mental health adverse events experienced by people which could inform risk mitigation procedures. (89%)
	Whether the event led to the person terminating therapy. (89%)
	A description of the adverse event (free text). (89%)
	Duration of adverse event. (89%)
	New mental health symptoms. (89%)
	Individuals are not aware that the Yellow Card Scheme is used to report the adverse reactions of digital mental health therapies. (83%)
	When categorising adverse events, a broad mental health category is needed with subtypes that relate to the different adverse events that could occur under that category. (83%)
Developing a harmonised approach for assessing the safety of face-to-face and DMHIs	**Having a harmonised approach for safety reporting across face-to-face and digital interventions will increase our knowledge of the risks of mental health therapies and contribute to stronger safety standards and safer interventions.** (94%)
	Using a harmonised approach to assess safety in face-to-face and digital mental health therapies would allow us to more easily compare the safety of face-to-face and digital therapies. (89%)
	If there was one structured way to report adverse events in face-to-face and digital mental health therapies, clinicians and mental health professionals would be more likely to report adverse events. (83%)
	Safety issues that have to do with clinical support need to be harmonised between face-to-face and DMHIs, because of their commonalities. In a digital context, safety issues that arise because of using a digital tool would be added to that. (83%)

### Enhancing the quality of adverse events data in DMHIs

Under this theme, experts echoed researchers’ findings on the poor quality of adverse events data in DMHI studies.^1^ A total of 94% of experts agreed that ‘The digital mental health field lacks clear guidance on what adverse events data should be collected, when and how this should happen, and how these should be categorised, analysed and reported’. All experts agreed that ‘Generating publication guidelines for reporting adverse events data that are required by journals can help ensure that adverse events data are collected and reported consistently’. Furthermore, under this theme experts concluded that ‘Determining the relatedness of the adverse event to the therapy is paramount for assessing safety’ (94%) and that ‘When assessing adverse events in digital mental health interventions, it is very important to differentiate between self-guided and clinician-guided therapies’ (89%).

### Redefining SAEs for DMHIs

One expert said in round 1: ‘The current definition of serious adverse events (hospitalisation, death, congenital abnormalities … ) is not fit for purpose for mental health interventions compared to pharmaceutical, surgical etc.’. When this item was later shared with the panel of experts in round 3, 94% concurred with it. In all, 83% of experts agreed that ‘Congenital abnormalities as a serious adverse event of digital mental health interventions is not plausible’. Experts also expressed that the traditional definition of an SAE is not comprehensive of what should be considered an SAE for a DMHI. All experts agreed that ‘In the context of mental health therapies, it is important for “serious” to be inclusive of serious effects on mood, behaviour, general well-being and functioning, not just physical harms’. In addition to loss of life, hospitalisation and disability, suicide attempts (94%), increase in harm towards others (100%), increase in intentional self-harm (100%) and seizures that are induced because of on-screen flashes (100%) should be considered SAEs even if they do not lead to hospitalisation. Experts added that in the case of a DMHI, any harm that is sustained for longer than 6 months should be considered an SAE (89%).

### Reassessing short-term deterioration in face-to-face and DMHIs as a therapeutic risk

Experts reached a consensus that ‘the short-term distress generated by focusing on the emotional experience (perhaps discussing painful or traumatic experiences or going into new or anxiety-provoking situations) is a part of the therapy process and should not be considered an adverse event or safety concern’ (89%). However, they also acknowledged that such a deterioration could lead to negative outcome/harm; thus, such deterioration needs to be monitored and not ignored (83%). For that, experts encouraged long-term follow-ups to differentiate between short-term and long-term deterioration (89%). They also suggested several ways to support individuals experiencing deterioration; eight of these reached consensus (see Supplementary Appendix C).

### Maximising the benefit of the Yellow Card Scheme in DMHIs

All experts agreed that ‘There is not enough awareness *among users* that the Yellow Card Scheme is used for reporting adverse reactions in digital mental health therapies’. Furthermore, 83% of experts agreed with the item ‘*I was not aware* that the Yellow Card Scheme is used to report the adverse reactions of digital mental health therapies’. Experts acknowledged that one drawback of the Yellow Card Scheme for collecting adverse reactions is that it relies on individuals to report risks, which they may not be willing or able to do; for that, they suggested that Patient Reported Outcomes could be automatically collected within the digital therapy itself to help identify risks (94%).

Acknowledging the benefit of the Yellow Card Scheme, experts determined a few improvements that would make it more useful for DMHIs. First, experts noted that grouping all mental health adverse reactions under ‘psychiatric disorders’ risks losing useful and important detail on the different mental health adverse reactions experienced by individuals and may discourage reporting milder mental health adverse reactions (89% and 94% respectively). Instead, they suggested having a broader and more inclusive category such as ‘mental health adverse reactions’, with subtypes that relate to the different adverse reactions that could occur under that category (94% and 83% respectively). Experts settled on four proposed subtypes: intentional self-harm (100%), threat to others (100%), suicidal ideation/behaviours (94%) and new mental health symptoms (89%). Additionally, experts agreed that having the following information about every mental health adverse reaction reported on the Yellow Card website under the DMHI's profile would be helpful: severity/impact on the individual (94%), duration of adverse reaction (89%), whether the event led to the individual terminating therapy (89%) and a description of the adverse reaction (free text) (89%).

### Developing a harmonised approach for assessing the safety of psychological interventions generally

Experts agreed that there should be a single harmonised approach for assessing and reporting the safety of face-to-face and DMHIs. Experts assented that such an approach would allow professionals to compare the safety of face-to-face and DMHIs (89%), and thus better understand the risks of mental health therapies, and contribute to stronger safety standards and safer interventions (94%). Moreover, experts speculated that ‘If there was one structured way to report adverse reactions in face-to-face and digital mental health therapies, clinicians and mental health professionals will be more likely to report them’ (83%). At the very least, the safety issues related to clinical support that are common to face-to-face and digital therapy such as the deterioration effect need to be harmonised, and the safety issues that arise from the use of a digital tool such as technical issues could be added to that (83%).

### Items with high disagreement rates

We also explored the items that had high rates of disagreement. See Supplementary Appendix B for the percentage of disagreement (i.e. rating disagree/strongly disagree) for all items. No items reached consensus disagreement (80% or higher). However, two items equalled or exceeded 50%. A total of 61% of experts disagreed that ‘triggering trauma-related flashbacks’ should be considered an SAE. Half of the experts disagreed that ‘triggering a panic attack’ should be considered an SAE. Although these items arose directly at the suggestion of some experts, more than half the group disagreed. These disagreement rates illustrate the difficulty defining what constitutes an SAE for DMHIs and reveal that some areas divide opinion.

## Discussion

This study successfully reached an experts’ consensus on ten recommendations for how to adapt the medical method used for assessing the safety of DMHIs. Our panel's consensus suggestions have the potential to improve the process of safety assessment, enhancing the field's approach to the safety of DMHIs. Table 3 provides a summary of the recommendations.

**Table 3:** List of recommendations on how to adapt the medical regulatory safety assessment model to meet the needs of the digital mental health field

Recommendation 1	Publish guidelines for professionals on how to assess and report adverse events data in digital mental health research.
Recommendation 2	Standardise the optimum way to assess the relatedness of an adverse event to the intervention, procedure or device, by using a variety of independent evidence sources.
Recommendation 3	When evaluating safety, differentiate between self-guided and clinician-guided interventions.
Recommendation 4	Broaden the scope of potential serious adverse events in digital studies to consider impact on mental health.
Recommendation 5	Monitor psychological deterioration to prevent potential harm, using post-treatment follow-ups.
Recommendation 6	Utilise the suggestions outlined (Supplementary Appendix C) to support people experiencing deterioration in any treatment context.
Recommendation 7	Improve the visibility of the Yellow Card Scheme as the primary means of reporting adverse reactions in the digital mental health field.
Recommendation 8	Change the Yellow Card ‘psychiatric conditions’ category label to ‘mental health adverse reactions’.
Recommendation 9 (a & b)	Expand the Yellow Card category that captures mental health data to incorporate informative subcategories and additional contextual information.
Recommendation 10	Develop an overarching, harmonised framework for assessing the safety of any psychological intervention (digital or otherwise).

### Improving adverse events data in DMHI trials

The Delphi study showed a consensus on the lack of clear guidelines on how to collect, analyse and report adverse events data in DMHIs. Using guidelines that were originally developed for pharmacological trials is adversely affecting the quality of adverse events data in DMHIs.^1,16^ Consequently, experts unanimously agreed that generating publication guidelines to support professionals on what adverse events data need to be collected, how often and how they need to be analysed, will help standardise adverse events reporting in the field and allow for more comprehensive and systematic analysis of adverse events data (*recommendation 1*).^13^

To effectively assess the safety of DMHIs, determining the relatedness of an adverse event to the treatment is key (*recommendation 2*), which can be less straightforward in a DMHI compared with a pharmacological intervention.^10^ Additional data collection to support this judgement could include onset, previous similar instances, and participant's and clinician's opinions on relatedness. This could improve the robustness of relatedness decision-making.^1^

When assessing or comparing the safety of DMHIs, it is important to differentiate between self-guided and clinician-guided DMHIs (*recommendation 3*). The concern is that self-guided interventions can pose more risk in the absence of a professional guiding the individual, explaining content to them, assessing risk and providing emotional support or encouragement to people when they need it.^1^ However, a review of the state of adverse events data in DMHIs found that studies of clinician-guided interventions were more likely to address adverse events in their publication compared with self-guided interventions.^1^ Adverse events in self-guided interventions are more likely to be missed because of the lack of human contact, and thus might be underreported. Thus, it is crucial for self-guided interventions to integrate within the product a way of collecting and monitoring adverse event and safety data, such as collecting pre/post data to detect deterioration or using automated triggers to alert clinicians to a worsening condition.^26^ In such cases, individuals would need to be informed and provide consent for such data to be collected and shared with the clinical team.

### Broadening the definition of SAE for DMHIs

Experts in this study agreed that the pharmacological frame of reference for SAEs is not ‘fit for purpose’ for DMHIs. They argued that the definition of an SAE in the digital mental health field is too narrow and needs to be inclusive of any potentially life-threatening effects on mood, behaviour, general well-being and functioning. They concurred that adverse events such as increase in harm towards others, increase in intentional self-harm and any harm that is sustained for longer than 6 months should be considered ‘serious’, regardless of whether it led to hospitalisation or not. An MHRA-led consultation on the proposed changes to the regulatory framework for medical devices in 2021 (891 respondents) found that professionals requested mental health impacts to be included under ‘serious’ adverse events.^27^ The UK government's response was that at that time ‘appropriate mechanisms were not in place to sufficiently regulate the inclusion of these impacts’.^27^ We therefore conclude that further consultation is needed to identify and operationalise how the existing SAE definition might best be broadened to capture serious impacts on mental health (*recommendation 4*).

### Short-term deterioration is not a safety concern in mental health therapy

Experts in this study reached a consensus that short-term deterioration of psychological symptoms occurring in the course of a psychological treatment can be an integral part of therapeutic change and is therefore not inevitably a safety concern. Nevertheless, experts stressed that these symptoms could amount to an adverse reaction, for example if the person drops out of therapy as a result. Thus, professionals need to monitor such deterioration and support individuals through it (*recommendation 5*). Similar to other negative effects, individuals need to be informed of the possibility of experiencing deterioration during therapy, and such deterioration needs to be normalised as part of the process.^28^ Additionally, guidelines for professionals and patients are needed on the management of short-term deterioration.^29^ Our experts provided a list of suggestions (see Supplementary Appendix C) for supporting people experiencing therapy-related deterioration.

### Adapting the Yellow Card Scheme for DMHIs

The medical field has already established that individuals are more likely than healthcare professionals to report adverse reactions that fall under the category ‘psychiatric conditions’.^18^ Experts in our study reached consensus on several ways to adapt the Yellow Card data collection and reporting process for DMHIs that are classified as medical devices, to maximise their benefit. First, there is a need for a campaign to raise awareness around the use of the Yellow Card Scheme for reporting the adverse reactions to DMHIs among both patients and professionals (*recommendation 7*). One suggestion is to make it a requirement for all DMHIs (including pre-market) to inform and direct people to the Yellow Card reporting website. Second, there was a consensus to use more appropriate, less stigmatising language by replacing ‘psychiatric disorders’ with ‘mental health adverse reactions’ (*recommendation 8*). Third, given that these are very general umbrella terms, it would be helpful to include further subcategories. Suggestions from our experts included ‘intentional self-harm’, ‘threat to others’, ‘suicidal ideation/behaviours’ and ‘new mental health symptoms’ (*recommendation 9a*). Fourth, it would be helpful to record the following contextual information for every reported adverse reaction: severity/impact on the individual, duration of adverse event, whether therapy was terminated as a consequence and a free text description of the adverse reaction (*recommendation 9b*).

### Using a harmonised approach to assess the safety of psychological therapies generally

Perhaps one of the reasons why it has been difficult to adapt the medical model for DMHIs is that this model was not developed to assess the safety of face-to-face psychological therapies. The current quality of adverse event reporting in trials of face-to-face interventions is poor and is not subject to the same regulation as medical devices.^12^ Face-to-face interventions would also benefit from a standardised safety assessment method. Currently there is insufficient information about how the negative effects or adverse events data of face-to-face and DMHIs compare.^13^ An ideal solution would be to develop one harmonised approach to assess the safety of face-to-face and DMHIs (*recommendation 10*). One harmonised approach could enhance the quality of safety assessments in traditional face-to-face interventions and ensure that safety evaluations of DMHIs are appropriate, proportionate and effective. Whether it is plausible to meet the needs of statutory regulators, clinicians, researchers, manufacturers and other stakeholders within a single overarching framework remains to be seen. Nevertheless, possible benefits our experts highlighted included better quality safety data, a more accessible method for non-medical mental health practitioners and better safety standards for both face-to-face and DMHIs.

### Limitations

There are several limitations of this Delphi study. First, patients’ and their carers’ perspectives were absent in the results. Second, 65% of experts who participated work in higher education, leading to a likely overrepresentation of views from within this sector. It is important to note that 9 out of the 16 invitees (56.25%) who were invited but did not participate in the study came from non-academic backgrounds, primarily clinicians and co-founders. Third, evidence reliability is not possible in Delphi studies as the results might have differed if a different group of experts had been selected.

## Conclusion

This Delphi study reached expert consensus on ten recommendations that can be used to adapt the medical regulatory safety assessment model to meet the needs of the digital mental health field. These recommendations, if implemented, have the potential to improve the process of safety assessment of psychological therapies in general and DMHIs in particular. In turn this may result in better quality of mental health safety data, a more effective risk mitigation process in DMHIs and more accurate risk–benefit analysis of DMHIs.

## Supporting information

Taher et al. supplementary material Taher et al. supplementary material

## Data Availability

The data that support the findings of this study are available on request from the corresponding author, J.Y.
