# Supplementary material for: Bridging the gap from medical to psychological safety assessment: consensus study in a digital mental health context
Source: BJPsych Open. 2024 Jun 3;10(4):e126. doi: 10.1192/bjo.2024.713 (PMC11363077; doi:10.1192/bjo.2024.713)
Supplement: Taher et al. supplementary material [file S2056472424007130sup001.docx]

## **Appendix A**

***List of Qualitative Questions – Round 1***

1. How familiar are you with the Yellow Card scheme? Did you know that it is used to report the side effects of digital mental health interventions?
2. What do you think should be considered a “serious” adverse event in a digital mental health intervention/therapy?
3. What are your thoughts on the current method used to assess risk in digital mental health interventions i.e., collecting and reporting adverse events data?
4. In your opinion what is the disadvantage of lumping all mental health risks under one category when assessing the safety of a digital mental health intervention?
5. How can this be improved? What other details do you think need to be reported?
6. In your view, what are the advantages and disadvantages of seeking a harmonized approach for safety reporting across face-to-face and digital mental health interventions?
7. In your view, can short term distress, or symptom deterioration be an integral or inevitable part of a mental health intervention/therapy?
8. If patients experience short-term deterioration during therapy and therapy is effective at the end of treatment, would you still see deterioration as a risk/side effect of the therapy? If not, how do you conceptualize it?
9. When assessing and reporting the possible risks of a digital mental health intervention, do you think it is important to differentiate between short-term deterioration that occurs during treatment, and long-term deterioration that lasts after therapy is complete? If so, why and how can that be done?
10. Let’s now assume for the sake of this survey that some risks of psychological therapies are integral to treatment. How can this risk be mitigated, or are patients supported to avoid drop-out and loss of potential therapeutic benefit for patients in face-to-face therapy?
11. How about in digital mental health interventions (self-guided or therapist guided)?

## **Appendix B**

## ***Full list of items generated in round 1 with corresponding consensus rates***

| **Item Number** | **Generated Item** | **% Agreement** | **% Disagreement** |
| --- | --- | --- | --- |
| Item 1 | The current method used for assessing the safety of digital mental health therapies focuses on the medical and physical impact of the therapy rather than the mental health one, which is more relevant in the case of digital mental health therapies. | 61 | 28 |
| Item 2 | Determining the relatedness of the adverse event to the therapy is paramount for assessing safety. | *94** | 6 |
| Item 3 | When assessing adverse events in digital mental health interventions, it is very important to differentiate between self-guided and clinician-guided therapies. | *89** | 0 |
| Item 4 | The digital mental health field lacks clear guidance on what adverse events data should be collected, when and how this should happen, and how these should be categorized, analysed and reported. | *94** | 0 |
| Item 5 | Symptom checklists are sometimes used to assess the safety of digital mental health interventions. If standardized, these would be helpful. | 72 | 0 |
| Item 6 | Generating publication guidelines for reporting adverse events data that are required by journals can help ensure that adverse events data are collected and reported consistently. | *100** | 0 |
| Item 7 | The current definition of serious adverse events (hospitalization, death, congenital abnormalities...) is not fit for purpose for mental health interventions compared to pharmaceutical, surgical etc. | *94** | 0 |
| Item 8 | When reporting serious adverse events, we need to consider the relatedness of the event to the treatment. A digital mental health intervention cannot directly lead to death or hospitalization, but it can lead to an increase in suicidal thoughts. | 72 | 17 |
| Item 9 | Congenital abnormalities as a serious adverse event of digital mental health intervention is not plausible. | *83** | 11 |
| Item 10 | In the context of mental health therapies, it is important for serious to be inclusive of serious effects on mood, behaviour, general well-being, and functioning not just physical harms. | *100** | 0 |
| Item 11 | Whether an adverse event is considered serious or not is dependent on the baseline experience of the individual. | 39 | 33 |
| Item 12 | Triggering trauma-related flashbacks | 22 | *61*** |
| Item 13 | Triggering a panic attack | 22 | *50*** |
| Item 14 | A suicide attempt | *94** | 0 |
| Item 15 | Seizures induced due to on-screen flashes | *100** | 0 |
| Item 16 | Worsening of mental health state that requires seeking help outside of the offered intervention | 78 | 11 |
| Item 17 | Any harm that is sustained for longer than six months | *89** | 0 |
| Item 18 | Increase in harm towards others | *100** | 0 |
| Item 19 | Triggering severe anxiety (pre-set threshold) | 67 | 6 |
| Item 20 | An increase in intentional self-harm | *100** | 0 |
| Item 21 | Deterioration of symptoms (pre-set threshold) | 50 | 17 |
| Item 22 | Increase in suicidal ideation | 67 | 6 |
| Item 23 | The short-term distress generated by focusing on the emotional experience (perhaps discussing painful or traumatic experiences or going into new or anxiety-provoking situations) is a part of the therapy process and should not be considered an adverse event or safety concern. | *89** | 11 |
| Item 24 | Symptom deterioration is normal and integral to psychological interventions. | 22 | 17 |
| Item 25 | Symptom deterioration is likely in therapy but not inevitable. | 78 | 0 |
| Item 26 | Symptom deterioration is not introduced to patients as a "side effect", rather as a normal part of the therapy process. | 72 | 0 |
| Item 27 | Short-term deterioration is an expected adverse event/side effect of therapy. | 56 | 0 |
| Item 28 | Whether symptom deterioration is an integral part of the treatment or not, will depend on the disorder. | 28 | 0 |
| Item 29 | If someone drops out of therapy because of deterioration, then it should be considered a side effect. | 22 | 6 |
| Item 30 | If there is a deterioration (albeit temporary) and it is clearly linked (related) to the intervention, then it should be considered a side effect. | 78 | 11 |
| Item 31 | The severity of the deterioration and its impact on the user's functioning is more important than its duration (short or long-term). | 50 | 0 |
| Item 32 | Short-term deterioration is a momentary side effect of therapy and one that should be monitored, so that negative outcomes/harm does not result from the treatment. | *83** | 0 |
| Item 33 | Long term follow ups are needed to differentiate between short term and long-term deterioration. | *89** | 0 |
| Item 34 | Short term deterioration may signal that there is something specific within the therapy that risks triggering deterioration. In that case more research is needed to determine whether that component is indeed risky, necessary for effectiveness, and can be deliverable in a safer way. | 72 | 0 |
| Item 35 | Patients need to be informed about short term symptom deterioration/distress (informed consent). In a digital context this can be replicated through text, audio, or video content. | *94** | 39 |
| Item 36 | It is important for therapists to normalize symptom deterioration. In a digital context, this can happen as part of a discussion with a trained mental health professional, or via the content of the digital therapy. | *94** | 0 |
| Item 37 | Develop and maintain a strong therapeutic alliance. | *94** | 0 |
| Item 38 | Monitoring of symptoms through regular symptom checks could also mitigate the risks of deterioration. | *83** | 0 |
| Item 39 | Digital mental health interventions should have pre-set thresholds of deterioration that trigger an escalation such as a face-to-face intervention to mitigate risk. | *83** | 0 |
| Item 40 | Discuss a clear rationale for each therapy activity/exercise. | *100** | 0 |
| Item 41 | Help the patient think about sources of support that they can use between sessions if needed. In a digital context, provide users with alternative sources of support should they need it. | *94** | 0 |
| Item 42 | Provide patients/users with information on how to manage symptom deterioration. | *94** | 0 |
| Item 43 | The yellow card scheme is well known in the UK as a way to report the adverse reactions of medications but not that of digital mental health therapies. | 67 | 6 |
| Item 44 | I was not aware that the yellow card scheme is used to report the adverse reactions of digital mental health therapies. | *83** | 22 |
| Item 45 | There is not enough awareness among users that the yellow card system is used for reporting adverse reactions in digital mental health therapies. | *100** | 6 |
| Item 46 | A key problem seems to be that it is relying on users to report risks, which they may not be willing or able to do. To mitigate that Patient Reported Outcomes could be automatically collected within the digital therapy itself. | *94** | 17 |
| Item 47 | Grouping all mental health adverse reactions under "psychiatric disorders" risks losing useful and important detail on the different mental health adverse events experienced by users which could inform risk mitigation procedures. | *89** | 6 |
| Item 48 | Grouping mental health adverse events under "psychiatric disorders" may discourage reporting milder mental health adverse events. Thus, using a broader category such as "mental health adverse events" is more appropriate. | *94** | 11 |
| Item 49 | When categorizing adverse events, a broad mental health category is needed with subtypes that relate to the different adverse events that could occur under that category. | *83** | 22 |
| Item 50 | Reliable deterioration as measured by reliable change index | 78 | 22 |
| Item 51 | New mental health symptoms | *89** | 28 |
| Item 52 | Suicidal ideation/behaviour | *94** | 0 |
| Item 53 | Intentional self-harm | *100** | 17 |
| Item 54 | Threat to others | *100** | 0 |
| Item 55 | Technical difficulties | 11 | 0 |
| Item 56 | Duration of adverse event | *89** | 6 |
| Item 57 | Severity/impact on the individual | *94** | 0 |
| Item 58 | Did the event lead to the patient terminating therapy | *89** | 0 |
| Item 59 | A description of the adverse event (free text) | *89** | 0 |
| Item 60 | Having a harmonized approach for safety reporting across face-to-face and digital interventions will increase our knowledge of the risks of mental health therapies and contribute to stronger safety standards and safer interventions. | *94** | 0 |
| Item 61 | Using a harmonized approach to assess safety in face-to-face and digital mental health therapies would allow us to more easily compare the safety of face-to-face and digital therapies. | *89** | 0 |
| Item 62 | If there was one structured way to report adverse events in face-to-face and digital mental health therapies, clinicians and mental health professionals will be more likely to report adverse events. | *83** | 0 |
| Item 63 | Safety issues that have to do with clinical support need to be harmonized between face-to-face and digital mental health interventions, because of their commonalities. In a digital context, the safety issues that would arise because of using a digital tool would be added to that. | *83** | 0 |
| Item 64 | The adverse events experienced by a user who is independently using a digital therapy are different to those in a face-to-face therapy. Thus, a harmonized approach to safety for digital and face-to-face mental health therapies may not be helpful. | 39 | 0 |

Notes

Agreement and disagreement rates reflect the percent of participants who agree/strongly agree and disagree/strongly disagree, respectively. ‘Neutral’ ratings are not included, meaning percents may not sum to 100 .

*indicates items reaching consensus, defined as 80% or more agreement rate. Dark green denotes agreement rate >90%; light green denotes agreement rate between 80 and 90%.

**indicates items reaching 50% or more disagreement.

## **Appendix C**

***List of suggestions, that reached consensus, on how to support users/patients who experience symptom deterioration during therapy.***

| **1** | Patients need to be informed about possible short-term symptoms deterioration/distress (informed consent). In a digital context this can be replicated through text, audio, or video content. |
| --- | --- |
| **2** | It is important for therapists to normalize symptom deterioration. In a digital context, this can happen as part of a discussion with a trained mental health professional, or via the content of the digital therapy. |
| **3** | Provide patients/users with information on how to manage symptom deterioration. |
| **4** | Monitoring symptoms through regular symptom checks could help mitigate the risks of deterioration. |
| **5** | Digital mental health interventions should have pre-set thresholds of deterioration that trigger an escalation such as a face-to-face intervention to mitigate risk. |
| **6** | Discuss a clear rationale for each therapy activity/exercise. |
| **7** | Develop and maintain a strong therapeutic alliance (in the context of both face-to-face and DMHI). |
| **8** | Help the patient/user to think about sources of support that they can use between sessions if needed. In a digital context, provide users with alternative sources of support should they need it. |
